# Adenosquamous carcinoma of the cervix: a case report from a resource-limited setting

**DOI:** 10.1097/RC9.0000000000000895

**Published:** 2026-08-21

**Authors:** Charles Malisaba Posite, Dieumerci Kaseso, Salomon Kabeza, Samuel Tumwesigire

**Affiliations:** a Department of Pathology, Kampala International University Western Campus, Ishaka, Uganda; b Department of Pathology, Université Catholique du Graben, Butembo, Democratic Republic of the Congo; c Department of Health, EarthRoost+, Beni, Democratic Republic of the Congo; d Department of Research, Inter-Sciences RDC, Butembo, Democratic Republic of the Congo; e Department of Gynecology and Obstetrics, Université Catholique du Graben, Butembo, Democratic Republic of the Congo; f Department of Gynecology and Obstetrics, Kampala International University Western Campus, Butembo, Democratic Republic of the Congo; g Department of Pathology, Mbarara University of Science and Technology, Mbarara, Uganda

**Keywords:** adenosquamous carcinoma, case report, cervix, resource-limited setting

## Abstract

**Introduction and importance:**

Adenosquamous carcinoma of the cervix (ASCC) is a rare, aggressive histological variant representing 1.8% to 5% of cervical malignancies. It is generally associated with a poorer prognosis than squamous cell carcinoma. In resource-limited settings, systemic barriers often preclude standardized multimodal management, necessitating management decisions shaped by limited resources. This case is presented following the SCARE criteria.

**Case presentation:**

A 64-year-old multiparous female presented to a tertiary hospital in Uganda with a multi-month history of dyspareunia and post-coital bleeding. Physical examination revealed an ulcerated cervical lesion; however, HPV DNA screening was unavailable. The patient underwent a total abdominal hysterectomy and bilateral salpingo-oophorectomy. Histopathology revealed a 3.0 cm mass with dual squamous and glandular differentiation, staged as pT1b1 Nx Mx according to the AJCC eighth edition. Due to a lack of equipment, radical lymphadenectomy and adjuvant radiotherapy were not performed. A 5-year surveillance plan was proposed for postoperative follow-up.

**Clinical discussion:**

This case highlights the gap between basic care and enhanced international standards for cervical malignancies in women. The Nx status reflects incomplete nodal staging in a resource-limited setting. Lack of equipment limited the ability to provide appropriate, screening-based surgical management, making prospective postoperative surveillance necessary.

**Conclusion:**

ASCC necessitates early detection and standardized multimodal care, objectives that are frequently precluded by infrastructure constraints in Sub-Saharan Africa. Improving outcomes for such aggressive histologies requires a concerted global effort to expand access to HPV vaccination, DNA-based screening, and equitable radiotherapy infrastructure. This may improve the likelihood of curative treatment in resource-constrained environments.

## Background

Cervical cancer remains a profound global health crisis, acting as a leading cause of oncological morbidity and mortality among women, with a disproportionate burden falling on women in low-resource settings^[^1^]^. While squamous cell carcinoma (SCC) is the most prevalent histological subtype, adenosquamous carcinoma of the cervix (ASCC) is a rare and clinically aggressive variant, representing approximately 1.8% to 5% of all cervical malignancies^[^2,3^]^.

HIGHLIGHTS

- A rare adenosquamous carcinoma was identified, showing dual squamous and glandular differentiation.
- Equipment gaps limited surgical options, precluding radical surgery.
- The lack of radiotherapy led to a planned postoperative surveillance approach.
- The case underscores the urgent need for equitable global access to HPV DNA-based screening.

As defined by the World Health Organization (WHO), ASCC is characterized by the simultaneous presence of both malignant squamous and glandular epithelial components^[^4^]^. This hybrid nature often translates to a more virulent clinical course compared to pure SCC or adenocarcinoma, frequently exhibiting higher rates of lymphovascular space invasion (LVSI) and a greater propensity for regional lymph node metastasis^[^5^]^. Consequently, ASCC is associated with a significantly higher mortality hazard and poorer 5-year survival rates, therefore requiring accurate staging and appropriate management^[^2,5^]^.

In many resource-constrained environments, the deficient access to radiotherapy and specialized surgical oncology services significantly compromises the standard of care. While the aggressive nature of ASCC is well-documented in high-resource settings, data remain limited on outcomes when radical surgery and nodal staging cannot be performed because of infrastructure gaps. This case addresses this gap by illustrating the management of ASCC through a basic level-of-care lens^[^1^]^. Furthermore, the absence of robust Human Papillomavirus (HPV) vaccination programs and standardized, DNA-based screening infrastructure leads to a high frequency of symptomatic, late-stage presentations^[^6–8^]^. Improving outcomes for patients with aggressive histologies like ASCC requires a concerted global effort to bridge the gap in screening access and radiotherapy infrastructure, highlighting the need for equitable access to oncologic care^[^1^]^. This report details a case of ASCC in a 64-year-old multiparous female from rural Uganda, highlighting the diagnostic challenges, surgical management, and the critical need for expanded oncological infrastructure in Sub-Saharan Africa (SSA), in line with the SCARE criteria^[^9^]^.

## Case presentation

In February 2026, a 64-year-old multiparous woman (P8 + 0) of Ankole ethnicity presented to the Department of Obstetrics and Gynecology at Kampala International University Teaching Hospital. The patient reported a multi-month history of dyspareunia, post-coital bleeding, and abnormal uterine bleeding. Her medical and family histories were unremarkable. Upon physical examination, a discrete ulcerated lesion was identified at the 6 o’clock position of the cervix, which exhibited contact bleeding, raising a high clinical suspicion of malignancy. Abdominopelvic ultrasonography was unremarkable, as were baseline laboratory investigations, including a complete blood count and Human Immunodeficiency Virus screening. Screening for HPV was not performed due to limited resources. Following a detailed discussion of the clinical findings, the patient consented to a total abdominal hysterectomy (TAH) with bilateral salpingo-oophorectomy (BSO).

**Figure 1.**
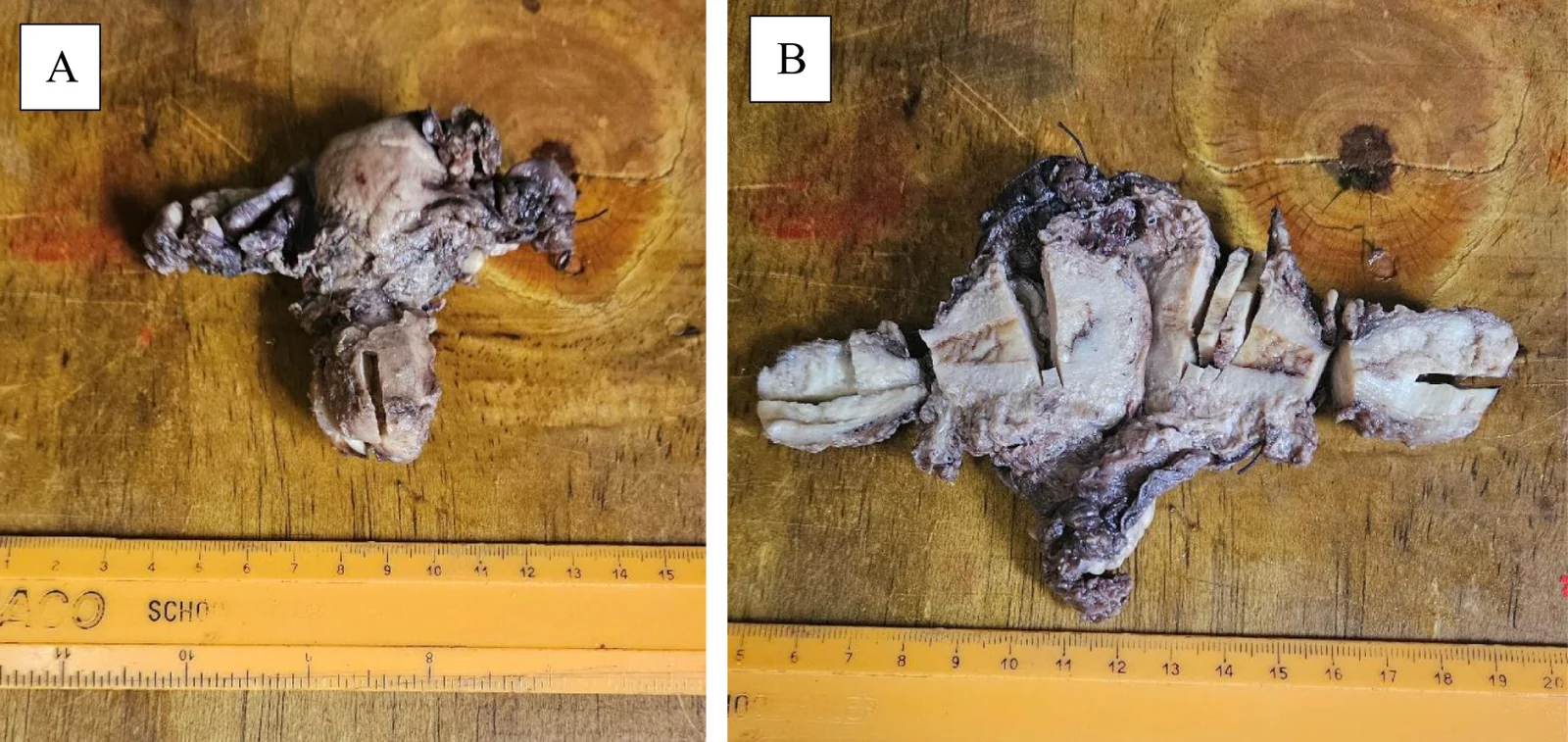
Total abdominal hysterectomy with bilateral salpingo-oophorectomy (A). The cut surface shows a mass involving the cervix (B).

The surgical specimen, weighing 90 grams, was submitted for histopathological analysis. Gross examination of the TAH + BSO specimen revealed a fundal-to-cervical length of 7.0 cm, with the cervix measuring 3.3 × 2.2 × 2.0 cm. The external cervical os was characteristic of a multiparous state and featured a 1.0 × 0.6 cm ulcerated area at the 6 o’clock position. Internal sectioning revealed an ill-defined mass measuring 3.0 × 2.0 × 2.0 cm extending along the cervical canal to the lower uterine segment. The endometrium, myometrium, and adnexa appeared grossly unremarkable (Fig. 1). No lymph nodes were received or retrieved for pathological assessment.

**Figure 2.**
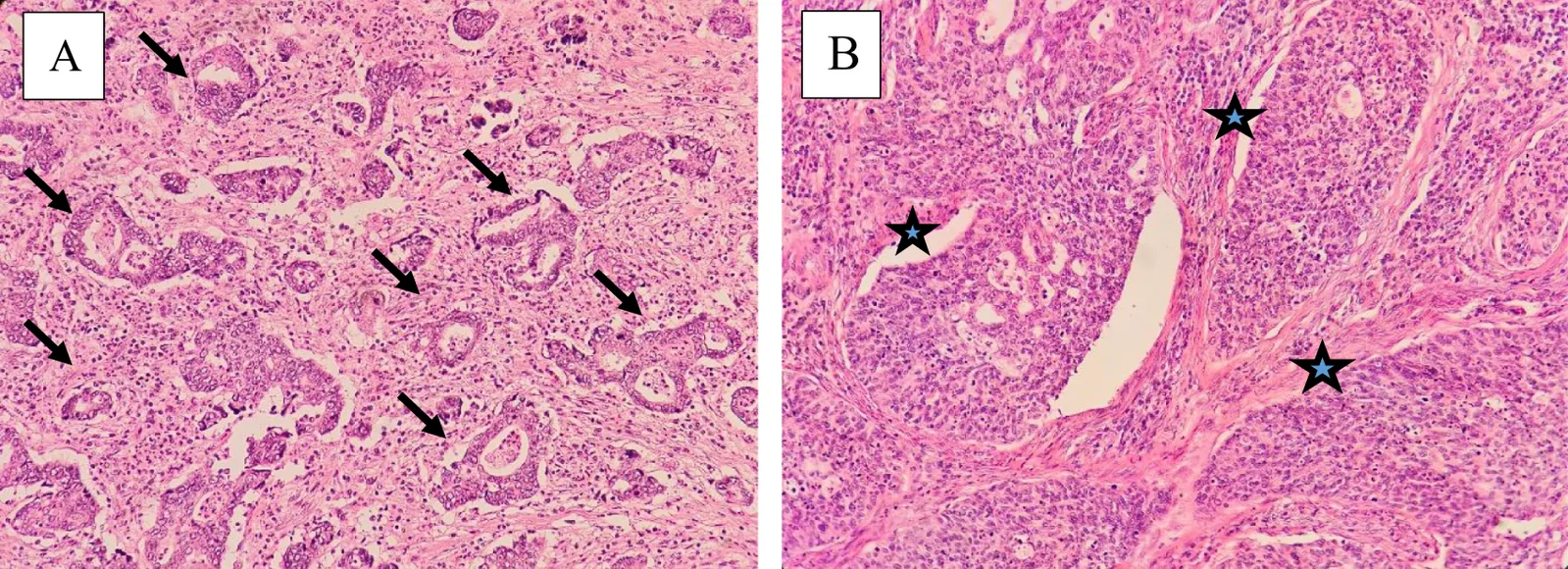
Histopathologic features of the neoplasm (H&E stain). (A) A section displaying glandular structures (arrows; × 100). (B) Pleomorphic cells with cellular atypia within nests of squamous tumor cells (stars; ×200). Both fields feature an infiltrate of lymphocytes and plasma cells within a desmoplastic stroma.

Microscopic evaluation of Hematoxylin and Eosin (H&E)-stained sections demonstrated cervical stromal infiltration comprising approximately 60% glandular and 40% squamous malignant components. This was accompanied by a prominent desmoplastic reaction and a mixed inflammatory infiltrate consisting of tumor-infiltrating lymphocytes and polymorphonuclear neutrophils (Fig. 2). While the ectocervical squamous epithelium exhibited focal areas of dysplasia, the endocervical surface lining remained unremarkable. Notably, the parametrium, lower uterine segment, myometrium, and adnexa were free of neoplastic involvement. Based on the histomorphological features, a diagnosis of ASCC was established and staged as pT1b1, Nx, Mx, reflecting tumor size and incomplete nodal and metastatic assessment.

The postoperative course was uneventful, and the patient was discharged on the eighth postoperative day. A proposed 5-year surveillance plan (Table 1) was outlined; only early postoperative review is currently available, and long-term outcomes are not yet known. It consisted of follow-up reviews every 4 months for the first year, transitioning to annual evaluations for a total of 5 years. Each review included a comprehensive clinical history and a thorough physical examination, including pelvic and rectal assessments.

**Table 1:** Timeline of events.

Time	Event	Outcome
15 February 2026	First appointment	–
	Abdominopelvic ultrasound scan	Unremarkable
	Blood grouping	O positive
	Complete blood count	Normal
	Admission	–
17 February 2026	Surgery + specimen submission for histopathology examination	TAH + BSO
25 February 2026	Pathology Report	ASCC
	Discharge	No complaint
Proposed 5-year surveillance schedule: Every 4 months for the first year, then annually up to 5 years	Planned history and physical examination, including pelvic-rectal examination, abdominal ultrasound scan, and chest X-ray	–

TAH, total abdominal hysterectomy; BSO, bilateral salpingo-oophorectomy; ASCC, adenosquamous carcinoma of the cervix.

## Discussion

The management of ASCC in a resource-limited setting like Uganda highlights a critical intersection between a rare, aggressive oncologic pathology and systemic healthcare barriers. ASCC is a distinct histological entity characterized by the simultaneous presence of both malignant squamous and glandular epithelial components. This hybrid nature typically translates to a more virulent clinical course and a poorer prognosis compared to pure SCC or adenocarcinoma^[^6^]^.

In this case, the diagnosis was established by the coexistence of these two malignant components within a heavily infiltrated cervical stroma. The WHO defines ASCC as containing a recognizable component of both adenocarcinoma and SCC. According to the American Joint Committee on Cancer (AJCC) eighth edition, the patient’s tumor was staged as pT1b1 Nx Mx^[^6,10^]^. The pT1b1 category refers to a clinically visible lesion confined to the cervix with a maximum dimension of ≤4.0 cm. The 3.0 cm mass described in this patient accurately fulfills these criteria. Nx indicates that regional lymph nodes could not be assessed. The AJCC stipulates that for an adequate pathological evaluation of regional lymph nodes (pN), the specimen should ordinarily include six or more lymph nodes. In this case, the Nx status reflects incomplete nodal staging in a resource-limited surgical setting because the lack of specialized gynecologic oncology support may preclude radical lymphadenectomy. Mx signifies that distant metastasis could not be clinically assessed^[^10^]^.

This case’s management highlights significant gaps between basic care provided in a resource-limited environment and the enhanced standards recommended by international oncology benchmarks^[^11^]^. In this clinical encounter, the surgical intervention was limited to a TAH and BSO, a basic approach necessitated by the absence of specialized gynecological oncology surgical support and radiotherapy infrastructure^[^12^]^. In contrast, an enhanced level of care could have consisted of radical hysterectomy paired with systematic pelvic lymphadenectomy and adjuvant cisplatin-based chemoradiotherapy. The gap in our case was clinically important. In fact, this may have left occult micrometastases undetected and untreated^[^12,13^]^. With resource-constrained surgical management, the patient’s care was limited. Thus, prospective postoperative surveillance came out mandatory and important, in default of definitive oncologic treatment.

The prognosis for ASCC is generally guarded, as the subtype is associated with higher mortality hazards and a greater propensity for regional lymph node metastasis compared with pure SCC. While LVSI does not change the T category in the AJCC system, its presence is a potent predictor of outcome and increases the probability of metastatic involvement of regional lymph nodes^[^3^]^. Because the hybrid nature of ASCC translates to a more virulent clinical course, the limited surgical approach used in this case, specifically the lack of nodal clearance, may increase the risk of undetected regional spread compared with enhanced standards of care^[^11,12^]^.

This case underscores the challenges posed by high-risk histologies in SSA. Given the aggressive nature of ASCC and the indeterminate nodal status (Nx), a proposed 5-year surveillance plan is appropriate. In regions with limited access to radiotherapy, clinicians often rely on surgery alone or suboptimal surveillance protocols. Because ASCC exhibits higher recurrence rates, meticulous pelvic and rectal examinations are imperative for early detection of potential vaginal vault or parametrial recurrence. In addition, abdominal ultrasound and chest X-ray may contribute to baseline postoperative assessment, although they do not substitute for full staging^[^14^]^.

Ultimately, improving survival for patients with aggressive histologies requires a concerted global effort to bridge the gaps in access to screening and in radiotherapy infrastructure. The lack of prior HPV screening and DNA testing increases the difficulty in managing this case, ranging from prevention to surgical management; this is a common pattern in regions where most global cervical cancer deaths occur^[^15^]^. In this context, the case underscores the urgent need for expanded access to HPV vaccination and DNA-based screening, which may help detect aggressive histologies like ASCC at an earlier, potentially more treatable stage, even within constrained clinical environments where radical surgery and radiotherapy remain inaccessible.

## Strengths and limitations

This report’s strength lies in its application of a resource-stratified framework to a rare, aggressive cervical histology, while its limitations include incomplete nodal staging, absent HPV DNA testing, lack of adjuvant radiotherapy, and limited generalizability as a single case.

## Conclusion

This case underscores the challenges of managing ASCC in a resource-limited healthcare environment. As an aggressive histological variant, ASCC necessitates early detection and standardized multimodal management, which are often hampered by infrastructure constraints in SSA. Limited access to preventive services and to timely diagnosis contributed to treatment constraints, specifically the omission of radical lymphadenectomy and adjuvant radiotherapy for a pT1b1 tumor. While the immediate postoperative course was favorable, the high-risk nature of ASCC and the Nx nodal status justify long-term surveillance. The case demonstrates the urgent need for expanded access to HPV vaccination and DNA-based screening to detect aggressive histologies such as ASCC at more curable stages.

## Data Availability

The data supporting the findings of this case report are included within the article. Any additional supporting information can be made available upon reasonable request from the corresponding author.
